# Effects of Dietary Supplementation of Bovine Lactoferricin on Rumen Microbiota, Lactation, and Health in Dairy Goats

**DOI:** 10.3389/fnut.2021.722303

**Published:** 2021-09-06

**Authors:** Yuexin Shao, Xian Zhang, Huawen Zhang, Bowen Tian, Yunan Weng, Jiangtao Huang, Christopher D. Lu, Huaiping Shi

**Affiliations:** ^1^Shaanxi Key Laboratory of Molecular Biology for Agriculture, College of Animal Science and Technology, Northwest A&F University, Yangling, China; ^2^College of Agriculture, Forestry and Natural Resource Management, University of Hawaii, Hilo, HI, United States

**Keywords:** lactoferricin, lactation, ruminal microorganisms, immunoglobulin A, goat

## Abstract

This study aimed to investigate the biological effects of supplementation of bovine lactoferricin (BLFc) at the rate of 100 mg/kg/day (LF-1) or 200 mg/kg/day (LF-2) in lactating dairy goats. Dietary BLFc supplementation increased the concentration of lactoferrin (LF) in the milk and serum (*p* < 0.05) without affecting the feed intake. In the LF-1 group, serum Fe, total antioxidant (T-AOC), and immunoglobulin A (IgA) were increased (*p* < 0.05), while malondialdehyde (MDA) was decreased (*p* < 0.05). In the LF-2 group, ruminal fluid pH value was decreased (*p* < 0.05), and the composition of ruminal microflora on day 42 was more diversified. *Firmicutes* phylum in the LF-2 group was the most abundant phyla. In contrast, *Bacteroidetes* phylum in the control group and the LF-1 group were the most abundant. Lower milk somatic cell count and higher IgA were observed in the LF-1 group and the LF-2 group than those in the control group (*p* < 0.05). These results suggested beneficial effects of supplementation of 100 mg/kg/day BLFc on reducing the oxidative stress and altering diversity of ruminal microflora.

## Introduction

Lactoferrin (LF) is highly expressed in the colostrum. Lactoferricin of ruminant is a peptide containing five tryptophan, three lysine, and several aromatic amino acid residues released from the N-terminal by pepsin hydrolysis under acidic conditions, with a molecular weight of approximately 3.1 KU (1). It is notable that bovine lactoferricin (BLFc) as an animal-derived antimicrobial peptide could induce beneficial health effects, that is, absorption of iron, antibacterial, anti-cancer, and immune ability and cell proliferation (2–4). Moreover, a recent study in piglets indicated that LFc could increase the antioxidant capacity and decrease diarrhea rate, suggesting the possible correlation between consumption of LFc and composition of gut microbiota (5–7). Rumen is a vital organ of nutrient digestion and metabolism and absorption in goats, and its microbial diversity will affect the health status of animals (8, 9). It was reported that the different compositions of ruminal microbiota were linked to the different levels of somatic cell count (SCC) and mastitis in ruminants (10, 11).

Recessive or subclinical mastitis is common in lactating dairy goats. The clinical manifestations include redness and swelling of the breast and elevated body temperature, and a sharp increase in the number of SCC. Goat milk fails to meet the national sales standards that possibly results in economic losses. When farmers use antibiotics without discretion, these antibiotics may easily lead to drug resistance and residue in milk. Therefore, there is a need for feed additives with no drug resistance, low residue, and low toxicity in combating recessive mastitis. Feed additives, such as micro preparations, probiotics, acid preparations, and antimicrobial peptides, decreased the risks of various diseases (12, 13). The BLFc used in this study belongs to antimicrobial peptides of animal origin. However, little information is available about how BLFc affects dairy goat health and reduces the rate of subclinical mastitis. We speculate that after the ingested BLFc reaching the rumen may interact with ruminal microbiota. The diverse and complex communities of microorganisms play important roles in animal health. We incorporated two different doses of BLFc into the diets of *Xinong Saanen* dairy goats to investigate the effects of BLFc on lactation performance, antioxidant ability, immune response, and rumen microbial composition.

## Materials and Methods

The experiment was carried out with the approval of the Animal Use and Care Committee of Northwest A&F University in China (No.IASCAAS-PG-39).

### Materials

Bovine lactoferricin was produced and supplied by DeYi Biotechnology., LTD (Hunan, China). The main component of BLFc is the product of chimeric expression of lactoferricin (BLF17-30) and lactoferrampin (LFAMPIN 265-284), two active regions of LF, with a total of 31 amino acid residues by genetic engineering technology. The amino acid sequence is consistent with the natural sequence, and its molecular weight is 3,953 Da.

### Animals, Experimental Design, and Diets

Twenty-one *Xinong Saanen* goats [60 ± 10 days in milk (DIM)] were used, and three BLFc treatments used were (a) control, fed the basal diet without BLFc supplementation; (b) orally supplemented with 100 mg/kg/day of BLFc (low dose, LF-1); and (c) orally supplemented with 200 mg/kg/day of BLFc (high dose, LF-2). BLFc supplements were thoroughly mixed with the concentrated feed and were fed first to goats to ensure complete consumption. After 2 weeks of adjustment phase, the experimental phased consisted of 42 days. All goats were housed indoors at Saanen Dairy Goat Farm of Northwest A&F University in China (Yangling, China; 34°16′56.24″N, +108°4′27.95″E). The temperature in the house was approximately 25 °C. The goats were fed basal diet, such as corn silage, a concentrate mixture, and alfalfa hay at 8:00 a.m. and 4:00 p.m. daily. The component of ration is seen in Supplementary Table 1. The feed intake of the goats was recorded daily during the 6-week experimental phase.

### Milk and Serum Analysis

Prior to oral supplementation of BLFc, jugular blood samples (5 ml) were collected (seven goats per treatment) on days 0, 14, 28, and 42. Blood samples were centrifuged at 3,500 rpm for 5 min and serum samples were stored at −80 °C. Seven goats per group were milked two times daily at 06:30 a.m. and 03:00 p.m., and AM and PM samples were taken and composited as daily samples. The composited milk samples were analyzed for milk fat, protein, lactose, and SCC (cells/ml) using a Combi 300 counter (Bentley, PA, USA). Additionally, LF and immunoglobulin A (IgA) in milk and serum were determined using ELISA kits (Jiangsu Meibiao Biotechnology Co. LTD, Nanjing, China). Serum Fe was analyzed by chromatometry (A039-1-1; Nanjing Jiancheng Bioengineering Institute, Nanjing, China). The malondialdehyde (MDA) of serum was measured using the thiobarbituric acid (TBA) method (A003-1-2; Nanjing Jiancheng Bioengineering Institute, Nanjing, China). Total antioxidant content (T-AOC) of serum was measured using a Total Antioxidant Capacity Assay Kit (A015-1; Nanjing Jiancheng Bioengineering Institute, Nanjing, China) based on the ferric reducing antioxidant power. The samples were detected at 520 nm, and the T-AOC was calculated according to the standard curve.

### Analysis of Rumen Microbial Diversity

At the end of the experimental phase, fresh rumen fluid samples were collected randomly *via* a stomach tube from three dairy goats of each group. Before sampling, the mouth of a goat was opened and then, the head of a sampler connected with a vacuum pump was inserted into the mouth of the goat along the esophagus. The sampling head is a hard plastic tube with 3 cm in diameter and 90 cm in length. The vacuum pressure was set at 65 kPa. To minimize saliva contamination, approximately 30 ml of rumen fluid was discarded before actual sample collection. Samples were immediately filtered through four-layer gauze. Rumen pH was measured immediately using a portable pH meter (Model PHB-4, Shanghai Leica Scientific Instrument Co., Ltd., Shanghai, China). Rumen fluid samples were placed in a 50 ml centrifuge tube and centrifuged at 9,500 rpm for 3 min. The V4 regions of bacterial 16 S rRNA genes were amplified. The bacterial sequences were amplified using primers 338F ACTCCTACGGGAGGCAGCAG and 806 R GGACTACHVGGGTWTCTAAT. The bacterial amplification mixture consisted of 0.8 μl (5 μM) of each primer, 10 ng template DNA, 4 μl 5 × Fast Path buffer, 2 μl 2.5 mM dNTPs, 0.4 μl FastPfu polymerase, 0.2 μL bovine serum albumin (BSA), and added ddH_2_O to 20 μl. Amplification was performed with initial denaturation at 95 °C for 3 min; 30 cycles of denaturation at 95 °C for 30 s, annealing at 50 °C for 30 s, and elongation at 72 °C for 45 s. PCR products were excised from 2% agarose gels and purified with a QIAquick Gel Extraction Kit (Qiagen, Venlo, The Netherlands). The remaining DNA was sent to Shanghai Majorbio Biological Technology Co. Ltd for sequencing using an Illumina MiSeqPE250 (Illumina, San Diego, CA, USA).

### Statistical Analysis

The data were analyzed using SPSS 19.0 statistical software (version 19, SPSS Inc, Chicago, IL, USA). Repeated measure analysis was used with all data presented as least squares means and pooled SEM. Significant differences were declared at *p* < 0.05. Spearman's correlation test was used to examine relationships between serum LF and milk LF concentrations. Meanwhile, the general linear model (GLM) was used to conduct multi-variance ANOVA for BLFc levels and feeding time. The differences between groups were discussed when there was significant interaction between the treatment factors. Rumen microbial diversity sequence reads were divided and aligned into operational taxonomic units (OTUs) with 97% sequence similarity. The highest abundance sequences were compared with template regions in the Greengenes database (Vsesion https://drive5.com/uparse/ 7.0), and used to acquire taxonomic information for each OTU and species composition. To obtain the species classification information corresponding to each OTU, the comparison database of 16S rRNA of bacteria and archaea was used (Silva, Release138 https://www.arb-silva.de). R software was used to analyze the microflora population structures (14).

## Results

### LF Levels

All goats remained healthy throughout the entire experiment with no adverse health effects from the supplementation of BLFc. Dietary supplementation of 100 or 200 mg/kg/day of BLFc did not affect daily feed intake (Supplementary Table 2, *p* > 0.05). At the beginning of the experiment, serum LF concentrations were not different (*p* > 0.05) among the three groups (Table 1). With the time elongation, BLFc supplementation increased LF concentration in the serum, with higher concentrations in LF-1 group on day 28 and LF-2 group on day 42 (Table 1, *p* < 0.05). Multivariate analysis of variance between the treatment times suggested that serum LF had significant differences among the three groups at the same treatment time. Serum LF concentrations of all 21 dairy goats trended higher in the experimental period, and both LF-1 group and LF-2 group affected the serum LF. We also observed the interaction between BLFc supplementation and time on serum LF (Table 1, *p* < 0.05). The results indicated that in LF-1 group and LF-2 group on day 42, there were 57.50 and 61.28% higher concentrations of LF than day 0 of the experiment, respectively. Correlation analysis indicated that serum LF was positively correlated with milk LF in LF-1 group and LF-2 group (Supplementary Figures 1, 2, *p* < 0.01).

**Table 1:** The effect of supplementation of bovine lactoferricin (BLFc) at the rate of 100 mg/kg/day (LF-1) or 200 mg/kg/day (LF-2) on the serum lactoferrin (LF) levels of dairy goats over time.

**Item**	**Time point (day)**	**Control**	**LF-1**	**LF-2**	**SEM**	***p-*** **value**		
						**Treatment**	**Time**	Treatment*Time
LF in the serum (μg/mL)	0	158.68^B^	160.24^B^	192.19^C^	8.67	0.211	–	–
	14	153.74^B^^c^	195.65^B^^b^	259.59^B^^a^	11.56	<0.01	0.029	<0.01
	28	151.64^B^^b^	246.97^A^^a^	252.46^B^^a^	12.29	<0.01	<0.01	<0.01
	42	198.00^A^^c^	252.77^A^^b^	309.98^A^^a^	13.62	0.01	<0.01	<0.01

abc *Treatment means with different letter differ (p < 0.05) in the same line*;

ABC *Treatment means with different letter differ (p < 0.05) in the same column*.

### Physiological Functions

Multivariate ANOVA between the treatment time and BLFc supplementation indicated that serum IgA was different among the three groups at the same treatment time. Although serum IgA of all 21 dairy goats trended higher in the experimental period, supplementation of BLFc increased serum IgA significantly (Table 2, *p* < 0.05). Serum IgA in the LF-1 group was higher than the control group (Table 2, *p* < 0.05). Within the same treatment time, differences in serum Fe were observed among three groups (Table 2, *p* < 0.05). Compared to the control group, higher serum Fe and T-AOC were observed in the LF-1 group and the LF-2 group on day 28 (Table 2, *p* < 0.05). Meanwhile, serum MDA in the LF-1 group was lower than that in the control group and the LF-2 group on day 28 (Table 2, *p* < 0.05). Multivariate analysis of treatment time and BLFc supplementation indicated that serum MDA was different among the three groups on day 28 (Table 2, *p* < 0.05). The LF-1 group on day 28 had 20.02, 64.02, and 70.41% higher concentrations of IgA, Fe, and T-AOC than at the onset of the treatment, respectively. Serum MDA at the same time was 59.39% lower than at the onset of the treatment in the LF-1 group.

**Table 2:** The effect of supplementation of BLFc at the rate of 100 mg/kg/day (LF-1) or 200 mg/kg/day (LF-2) on IgA, Fe, total antioxidant content (T-AOC), and Malondialdehyde (MDA) in the serum of dairy goats.

**Item**	**Time point (day)**	**Control (*n* = 7)**	**LF-1 (*n* = 7)**	**LF-2 (*n* = 7)**	**SEM**	***p-*** **value**		
						**Treatment**	**Time**	Treatment*Time
IgA in the serum (μg/mL)	0	428.01^BC^^b^	438.47^B^^b^	487.15^B^^a^	9.77	0.021	–	–
	14	408.96^C^^b^	504.55^A^^a^	556.72^A^^a^	17.25	<0.01	0.057	<0.01
	28	488.52^A^^b^	526.23^A^^ab^	573.25^A^^a^	14.31	<0.01	<0.01	<0.01
	42	468.31^AB^^b^	532.35^A^^a^	574.15^A^^a^	15.17	<0.01	<0.01	<0.01
Fe (μmol/L)	0	69.73^B^	83.02^B^	65.66^B^	9.50	0.749	–	–
	14	124.53^A^	111.96^AB^	109.76^A^	5.55	0.524	<0.01	0.018
	28	88.59^AB^^b^	136.17^A^^a^	145.36^A^^a^	10.35	0.032	<0.01	<0.01
	42	101.84^AB^	123.42^A^	112.68^A^	8.88	0.645	<0.01	0.092
T-AOC (U/mL)	0	1.04^B^	0.98^B^	1.20	0.12	0.730	–	–
	14	1.55^A^	1.47^AB^	1.30	0.08	0.479	0.096	<0.01
	28	1.09^B^^b^	1.67^A^^a^	1.67^a^	0.10	0.014	0.113	<0.01
	42	1.34^A^	1.69^A^	1.57	0.08	0.630	0.073	<0.01
MDA (nmol/mL)	0	9.08^BC^	12.14^A^	8.81^AB^	0.79	0.159	–	–
	14	6.12^C^	6.84^B^	5.88^B^	0.48	0.728	0.045	<0.01
	28	10.20^B^^a^	4.93^B^^b^	10.12^AB^^a^	0.91	0.018	0.203	0.016
	42	14.00^A^	10.44^A^	10.93^A^	0.95	0.257	0.159	0.130

ab *Treatment means with different letter differ (p < 0.05) in the same line*;

ABC *Treatment means with different letter differ (p < 0.05) in the same column*.

### Ruminal Microorganisms

**Figure 1:**
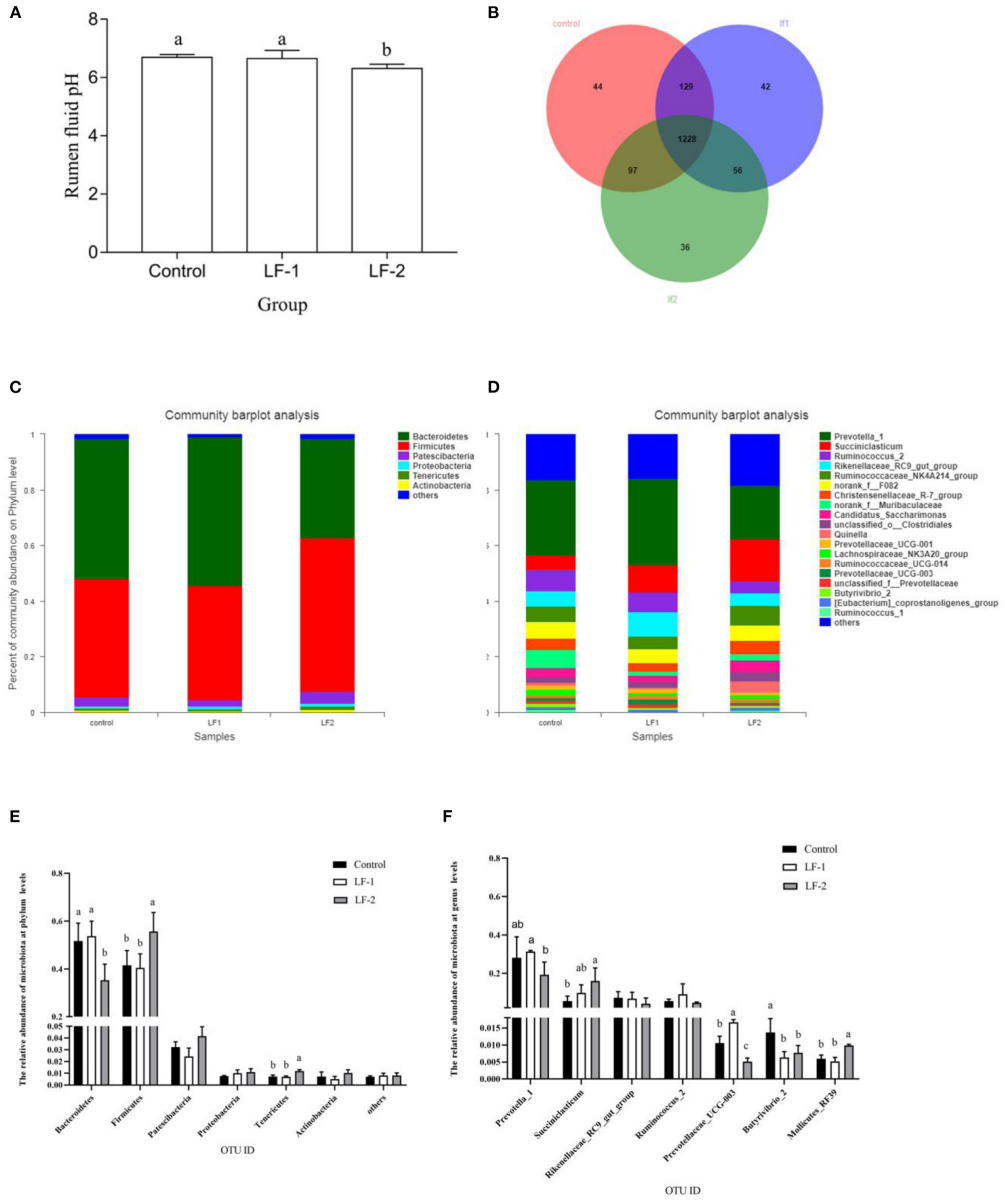
The effect of supplementation of BLFc at the rate of 100 mg/kg/day (LF-1) or 200 mg/kg/day (LF-2) on **(A)** rumen pH, **(B)** number of operational taxonomic unit (OTU), **(C)** rumen microbial diversity at the phylum level, **(D)** rumen microbial diversity at the genus level in dairy goats, **(E)** effects of BLFc on the relative abundance of rumen microbiota at phylum levels in goats, and **(F)** effects of BLFc on the relative abundance of rumen microbiota at genus levels in goats. In **(A)**, **(E)**, and **(F)**: ^abc^Treatment means with different letter differ (*p* < 0.05).

Rumen pH in the LF-2 group was lower than that in the LF-1 group and the control group (Figure 1A, *p* < 0.05). There was no difference in pH value between the LF-1 group and the control group (Figure 1A, *p* > 0.05). The sequencing data, based on the principle that the similarity is >97%, were divided into OTUs. With the removal of low-quality reads from sequencing data, we obtained 326,508 total sequences for bacteria, with an average of 100,825 sequences per sample. There were 1,498, 1,455, and 1,417 OTUs in the control group, LF-1 group, and LF-2 group, respectively. The three groups had a total of 1,228 OTUs (Figure 1B).

Goat rumen was found to be dominated by two bacterial phyla, *Bacteroidetes* and *Firmicute*s, while other phyla, such as *Patescibacteria, Proteobacteria, Tenericuts*, and *Actinobacteria* were less in numbers (Figure 1C). At the phylum level, *Bacteroidetes* were significantly decreased and *Firmicutes* were significantly increased in LF-2 group compared with the control group and the LF-1 group (Figures 1C,E). The three most abundant phyla in LF-1 group and the control group were *Bacteroidetes, Firmicutes*, and *Patescibacteria*. However, *Firmicutes* in LF-2 group was the most abundant (Figures 1C,E). At the genus level, the average relative abundance of *Prevotella 1* gut in LF-1 group was higher (*p* < 0.05) than that in LF-2 group (Figures 1D,F). The relative abundance of *succiniclasticum* in LF-2 group was higher (*p* < 0.05) than that in the LF-1 group and the control group (Figures 1D,F).

### Performance of Lactating Goats

There was no difference in milk yield among the three groups (Table 3, *p* > 0.05), with a trend of higher milk yield in BLFc-treated group on day 42. The results showed that the control group had 17.59% lower milk production at the end of the 6-week treatment (Table 3, *p* < 0.05). Neither the LF-1 group nor the LF-2 group showed any effect on the milk yield greatly (Table 3, *p* > 0.05). However, the supplementation of BLFc affected the milk composition. Milk fat in LF-2 group on day 28 was lower than that in the control group and LF-1 group (Table 4, *p* < 0.05).

**Table 3:** The effect of supplementation of BLFc at the rate of 100 mg/kg/day (LF-1) or 200 mg/kg/day (LF-2) on milk production throughout the experiment.

**Time**	**Control**	**LF-1**	**LF-2**	**SEM**	***p-*value**
Average milk production per day of the first week	2.16^A^	2.13^A^	1.95	0.06	0.488
Average milk production per day of the second week	1.91^B^	1.95^B^	1.86	0.05	0.769
Average milk production per day of the third week	2.08^A^	2.06^A^	1.87	0.07	0.484
Average milk production per day of the fourth week	1.93^B^	2.07^A^	1.94	0.08	0.696
Average milk production per day of the fifth week	1.79^C^	2.01^A^	1.87	0.08	0.474
Average milk production per day of the sixth week	1.78^C^	2.06^A^	1.94	0.08	0.319

ABC *Treatment means with different letter differ (p < 0.05) in the same column*.

**Table 4:** The effects of supplementation of BLFc at the rate of 100 mg/kg/day (LF-1) or 200 mg/kg/day (LF-2) on the milk composition in dairy goats.

**Time point (day)**	**Item**	**Control**	**LF-1**	**LF-2**	**SEM**	***p*-value**
0 d	Fat (%)	3.73 ± 1.47	4.37 ± 1.96	3.94 ± 1.54	0.36	0.78
	Protein (%)	3.45 ± 0.54	3.28 ± 0.15	3.08 ± 0.27	0.08	0.20
	Lactose (%)	5.02 ± 0.26	5.05 ± 0.21	4.92 ± 0.26	0.53	0.56
14 d	Fat (%)	4.26 ± 0.63	4.30 ± 0.76	3.65 ± 0.61	0.15	0.16
	Protein (%)	3.41 ± 0.36	3.24 ± 0.23	3.17 ± 0.21	0.06	0.26
	Lactose (%)	4.95 ± 0.27	4.99 ± 0.22	4.94 ± 0.27	0.53	0.94
28 d	Fat (%)	4.21 ± 0.86^a^	4.18 ± 0.60^a^	3.30 ± 0.50^b^	0.17	0.03
	Protein (%)	3.43 ± 0.46	3.22 ± 0.18	3.08 ± 0.20	0.07	0.12
	Lactose (%)	4.77 ± 0.16	4.76 ± 0.15	4.78 ± 0.14	0.03	0.97
42 d	Fat (%)	4.19 ± 0.92	4.17 ± 0.71	3.46 ± 0.59	0.17	0.15
	Protein (%)	3.44 ± 0.71	3.10 ± 0.22	2.95 ± 0.24	0.10	0.14
	Lactose (%)	4.66 ± 0.28	4.85 ± 0.11	4.76 ± 0.28	0.05	0.35

ab *Treatment means with different letter differ (p < 0.05) in the same line*.

### Bioactive Substances in Milk

Milk SCC was reduced in the LF-1 and LF-2 groups. The SCC in the LF-1 group and the LF-2 group was lower compared with the control group on day 42 (Table 5, *p* < 0.05). Milk IgA concentrations in goats received that BLFc supplementation were higher than the control group on day 42 (Table 6, *p* < 0.05). Milk IgA in the LF-2 group was the highest among the three groups (Table 6, *p* < 0.05). Milk LF concentrations in goats that received BLFc supplementation were higher than that in the control group, and in the LF-2 group being the highest (Table 6, *p* < 0.05). Moreover, the results showed that there was a trend of higher IgA and LF in milk in the LF-1 and LF-2 groups. The LF-1 and LF-2 groups on day 42 had 45.20 and 29.55% higher concentrations of IgA in the milk than at the beginning of the treatment, respectively. The LF-1 and LF-2 groups on day 42 had 60.66 and 39.45% higher concentrations of LF in the milk than at the beginning of the treatment, respectively. There were the interactions between BLFc-treated concentration and BLFc-treated time on both IgA level and LF level in the milk of dairy goats (Table 6, *p* < 0.05).

**Table 5:** Effects of supplementation of BLFc at 100 mg/kg/day (LF-1) or 200 mg/kg/day (LF−2) on milk somatic cell counts (SCCs) (×1,000/ml) in dairy goats.

**Item**	**Time point (day)**	**Control**	**LF-1**	**LF-2**	**SEM**	***p-*value**
Milk somatic cell counts (×1,000/mL)	0	304.14	272.75	270.38	44.69	0.950
	14	225.50	151.13	163.60	51.09	0.832
	28	483.80	366.29	242.25	73.17	0.447
	42	407.38^a^	169.63^a^	63.83^b^	53.34	0.019

ab *Treatment means with different letter differ (p < 0.05) in the same line*.

**Table 6:** Effects of supplementation of BLFc at 100 mg/kg/day (LF-1) or 200 mg/kg/day (LF−2) on milk IgA and lactoferrin in dairy goats.

**Item**	**Time point (day)**	**Control**	**LF-1**	**LF-2**	**SEM**	***p-*** **value**		
						**Treatment**	**Time**	Treatment*Time
IgA in milk (μg/mL)	0	227.20^AB^	220.93^C^	238.29^B^	8.14	0.70	–	–
	14	205.43^B^^b^	257.84^C^^a^	288.17^A^^a^	11.06	<0.01	0.122	<0.01
	28	236.13^AB^^b^	294.60^AB^^a^	322.23^A^^a^	11.17	<0.01	<0.01	<0.01
	42	250.35^A^^b^	320.79^A^^a^	308.70^A^^a^	10.49	<0.01	<0.01	<0.01
LF in milk (μg/mL)	0	273.48^B^^b^	283.82^C^^ab^	343.12^C^^a^	13.16	0.058	−	−
	14	290.34^B^^b^	356.17^B^^a^	404.90^BC^^a^	14.92	<0.01	0.029	<0.01
	28	365.51^A^^b^	450.63^A^^a^	439.30^AB^^a^	12.92	<0.01	<0.01	<0.01
	42	382.96^A^^b^	458.99^A^^a^	478.47^A^^a^	12.73	<0.01	<0.01	<0.01

ab *Treatment means with different letter differ (p < 0.05) in the same line*;

ABC *Treatment means with different letter differ (p < 0.05) in the same column*.

## Discussion

This study suggested that BLFc decreased the rumen pH value and altered the composition of rumen microbiota. Higher dose of BLFc appeared to increase the ratio of *Firmicutes* phylum to *Bacteroidetes* phylum and decreased the abundance of *Prevotella* genus. The phylum of *Bacteroidetes* and *Firmicutes* were the most abundant phylum in rumen of the ruminant animal, and *Prevotella* is the most abundant genus. The increased ratio of *Firmicutes* phylum to *Bacteroidetes* phylum (F/B) will result in an increase in energy availability from the feed. The abundance of *Firmicutes* is associated with the degradation of fermentable carbohydrates (15). It is known that *Bacteroidetes* phylum is predominantly Gram-negative bacteria and sensitive to the change of environmental pH value (16). We also observed that *Butyrivibrio* genus and *Succiniclasticum* genus were depleted in BLFc-treated goats. It implied that BLFc altered the type of rumen fermentation. Several studies showed that the diversity and structure of ruminal microbiota affected the immune system (17–19). Antimicrobial peptides promote the abundance of *Firmicutes* and *Tenericutes* and increase LF synthesis in the peripartum period (20, 21). The previous research found that adding antimicrobial peptides to the diet reduced the pH value in the intestine and increased the abundance of *Firmicutes* such as lactic acid bacteria (6, 22).

It is noteworthy that the abundance of *Butyrivibrio* genus related to butyric acids is decreased in the present study, and there was an upper trend in the milk yield at the end of the experiment in goats that received the supplementation of BLFc. The decrease of *Butyrivibrio* genus reduced the formation of butyric acids and finally affected the milk fat (23), the last result of which has been observed in this study. In addition, some research certified that the formation of propionic acid in goat rumen is increased with the decrease of butyric acids (24–26). The high levels of propionic acid greatly improve the digestion and absorption of concentrated feed and furthermore increase the lactating performance of dairy goat, so we found that the milk yield in the dairy goat is improved in this experiment. The results demonstrated that BLFc supplementation in diet possibly alters the composition of rumen microbiota, with the strong alterations observed in the LF-2 group.

It has been reported that the levels of IgA and LF were related to SCC in the milk, and could be useful indicators for the detection of subclinical mastitis (27). We speculated that BLFc promoted lymphocyte differentiation, reduced the number of neutrophils, and enhanced the innate and adaptive immune (28). Although SCC in goats with BLFc supplementation was statistically different compared with the goats in the control group, the average SCC of goats with BLFc supplementation on day 42 was greatly lower than that in the control group (*p* < 0.05). The SCC represents the total cell count in goat milk, mostly are immune cells, such as macrophages, neutrophils, and lymphocytes (29). IgA could be effective to combat bacteria, such as *Staphylococcus aureus* and *Streptococcus agalactiae* infecting mammary epithelial cells (30). We observed that BLFc supplementation to the goats could increase the levels of LF in serum and milk and IgA in milk. Although the levels of LF in serum and milk were affected by the time and treatment interactions, the effect of BLFc supplementation over time was apparent. This suggests that LF supplementation at both levels could effectively increase the levels of LF in serum and milk, and the absorption and utilization of added BLFc by dairy goats was effective. These studies suggested that BLFc had a positive role on immune regulation. The LF is capable of passing through the blood/milk barrier (31–33) and plays a role in protecting the mammary gland from bacterial invasion.

Furthermore, the LF is a non-heme-iron glycoprotein that can promote the absorption and storage of iron (34). Iron, as an important constituent of hemoglobin, can improve the antioxidant capacity in serum and enhance cellular immunity. In the present study, there were elevated serum iron and T-AOC, and lower MDA level in goats with BLFc supplementation compared with the control group on day 28, which implied that BLFc possibly promoted the absorption of iron and oxidizing ability. These findings demonstrated that BLFc as an antioxidant will scavenge hydroxyl radicals and reduce the damage of active oxygen to the body. It was similar to the reported research that BLFc has many protective effects, such as reducing the levels of MDA, catalase, and glutathione in the serum and enhancing the antioxidant capacity in lambs (35).

## Conclusion

The study provided important evidence that 100 mg/kg/day BLFc supplementation to the diet of lactating dairy goats is capable of decreasing oxidative stress and inhibiting inflammation.

## Data Availability Statement

The datasets presented in this study can be found in online repositories. The names of the repository/repositories and accession number(s) can be found below: NCBI BioProject, PRJNA742184.

## Ethics Statement

The animal study was reviewed and approved by the experiment was carried out with the approval of the Animal Use and Care Committee of Northwest A&F University in China (No.IASCAAS-PG-39). Written informed consent was obtained from the owners for the participation of their animals in this study.

## Author Contributions

HS and CL conceived and designed the experiments. YS, XZ, HZ, BT, YW, and JH performed the experiments. YS analyzed the data. YS and HS wrote the paper. YS and CL helped perform the analysis and with constructive discussions. All authors read and approved the final manuscript.

## Conflict of Interest

The authors declare that the research was conducted in the absence of any commercial or financial relationships that could be construed as a potential conflict of interest.

## Publisher's Note

All claims expressed in this article are solely those of the authors and do not necessarily represent those of their affiliated organizations, or those of the publisher, the editors and the reviewers. Any product that may be evaluated in this article, or claim that may be made by its manufacturer, is not guaranteed or endorsed by the publisher.
