# Supplementary material for: Effects of Dietary Supplementation of Bovine Lactoferricin on Rumen Microbiota, Lactation, and Health in Dairy Goats
Source: Front Nutr. 2021 Sep 6;8:722303. doi: 10.3389/fnut.2021.722303 (PMC8450446; doi:10.3389/fnut.2021.722303)
Supplement: Supplementary file 1 [file Data_Sheet_1.docx]

Supplementary Material

**Supporting Table 1.**

Ingredients and Chemical Composition of the Experimental Diet.

| Item | Ingredients | g/kg |
| --- | --- | --- |
|  | Maize silage | 319 |
|  | alfalfa hay | 211 |
| concentrate mixture | maize grain | 286 |
|  | wheat bran | 67 |
|  | soybean meal | 62 |
|  | rapeseed meal | 42 |
|  | mineral and vitamin premix | 6 |
|  | calcium hydrogen phosphate | 5 |
|  | salt | 2 |
| chemical composition | crude protein (CP) | 113 |
|  | ether extract (EE) | 23 |
|  | nitrogen-free extract (NFE) | 373 |
|  | Ca | 10 |
|  | P | 5 |
|  | NEL (MJ/kg of DM) | 4.89 |

**Supporting Table 2.**

Effects of supplementation of lactoferricin at 100 mg/kg/d (LF-1) or 200 mg/kg/d (LF-2) on feed intake (DM, kg/d) in Saanen goats.

| Time | Control | LF-1 | LF-2 | SEM | *p-*value |
| --- | --- | --- | --- | --- | --- |
| Average intake per day of the first week | 30.51 | 30.17 | 29.83 | 0.36 | 0.763 |
| Average intake per day of the second week | 29.91 | 29.75 | 30.06 | 0,25 | 0.891 |
| Average intake per day of the third week | 30.63 | 30.98 | 30.52 | 0.32 | 0.849 |
| Average intake per day of the fourth week | 29.91 | 30.65 | 29.09 | 0.20 | 0.873 |
| Average intake per day of the fifth week | 27.30 | 28.13 | 27.23 | 0.37 | 0.571 |
| Average intake per day of the sixth week | 27.70 | 26.8 | 26.28 | 0.28 | 0.438 |


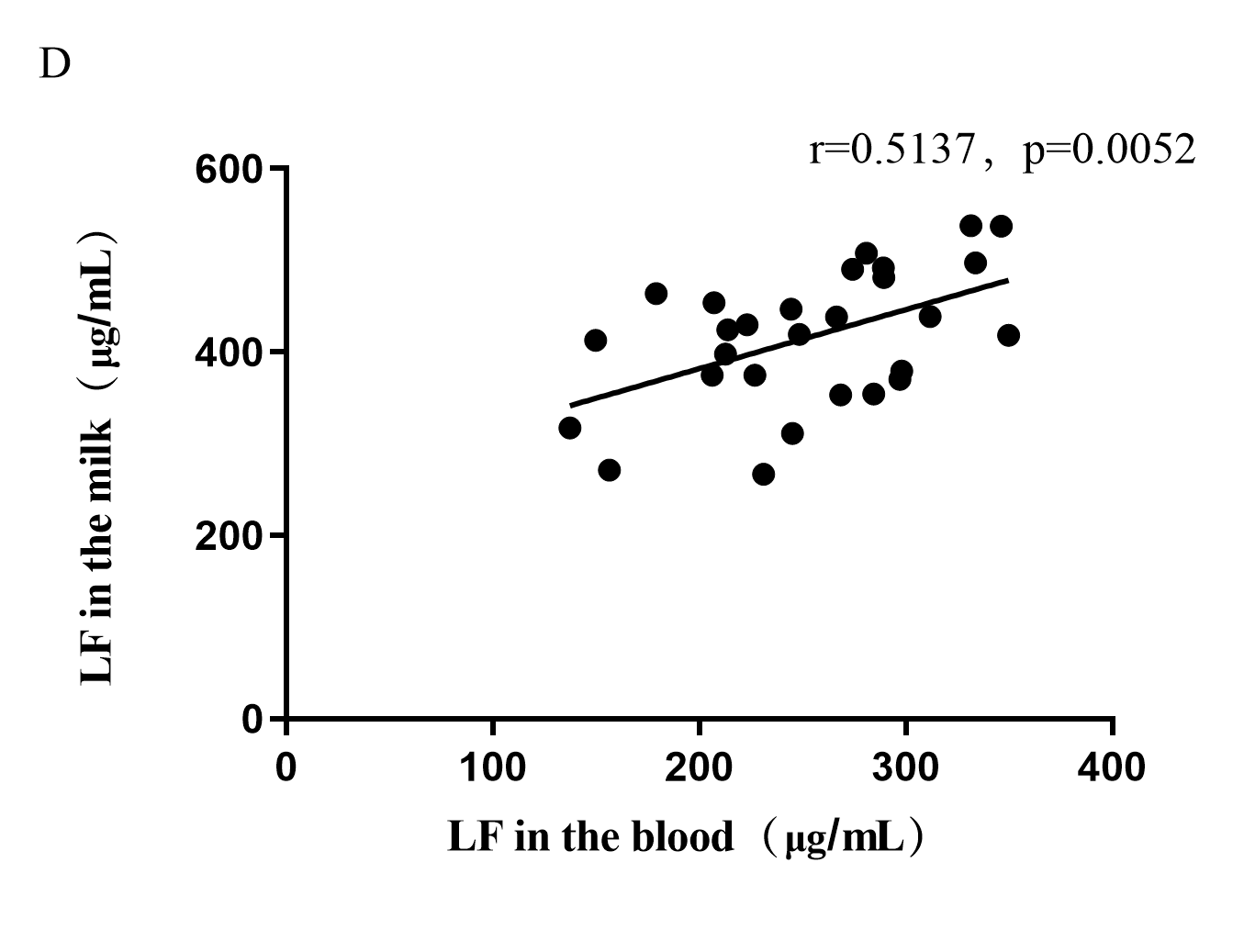


**Supporting Figure 1.** Correlation analysis of serum LF correlated with milk LF in LF-1 group.


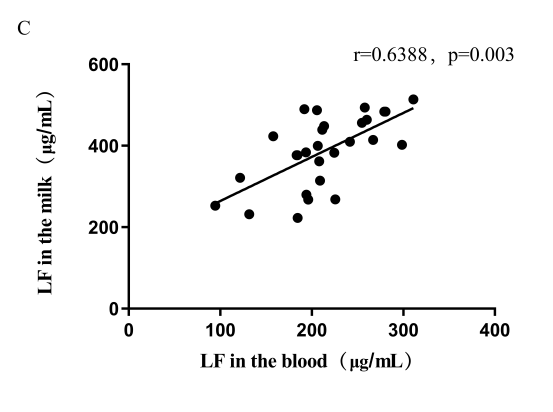


**Supporting Figure 2.** Correlation analysis of serum LF correlated with milk LF in LF-2 group.

**
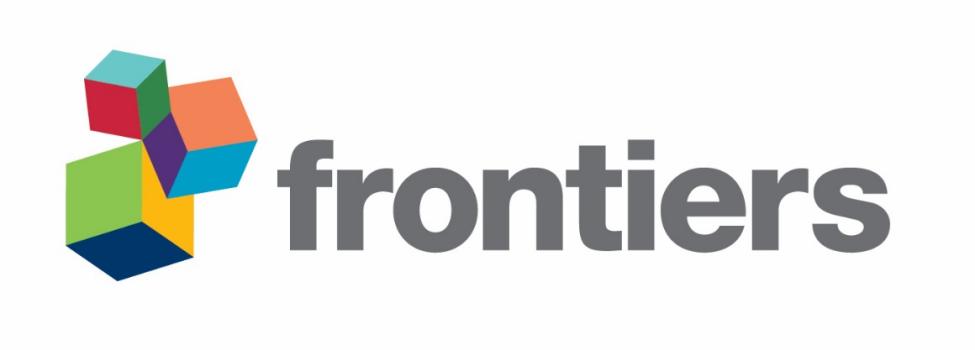
**
